# On the human health benefits of microalgal phytohormones: An explorative *in silico* analysis

**DOI:** 10.1016/j.csbj.2023.01.032

**Published:** 2023-01-25

**Authors:** Angelo Del Mondo, Annamaria Vinaccia, Luigi Pistelli, Christophe Brunet, Clementina Sansone

**Affiliations:** Stazione zoologica Anton Dohrn, sede Molosiglio Marina Acton, via ammiraglio F. Acton, 55, 80133 Napoli, Italy

**Keywords:** Signaling, Target fishing, Auxins, Cytokinins, Gibberellins, Brassinosteroid

## Abstract

Phytohormones represent a group of secondary metabolites with different chemical structures, in which belong auxins, cytokinins, gibberellins, or brassinosteroids. In higher plants, they cover active roles in growth or defense function, while their potential benefits for human health protection were noted for some phytohormones and little explored for many others. In this study, we developed a target fishing strategy on fifty-three selected naturally occurring phytohormones covering different families towards proteins involved in key cellular functions related to human metabolism and health protection/disease. This *in silico* analysis strategy aims to screen the potential human health-driven bioactivity of more than fifty phytohormones through the analysis of their interactions with specific targets. From this analysis, twenty-eight human targets were recovered. Some targets e.g., the proteins mitochondrial glutamate dehydrogenase (GLUD1) or nerve growth factor (NGF) bound many phytohormones, highlighting their involvement in amino acid metabolism and/or in the maintenance or survival of neurons. Conversely, some phytohormones specifically interacted with some proteins, e.g., SPRY domain-containing SOCS box protein 2 (SPSB2) or Inosine-5′-monophosphate dehydrogenase 1 (IMPDH1), both involved in human immune response. They were then investigated with a molecular docking analysis approach. Our bioprospecting study indicated that many phytohormones may endow human health benefits, with potential functional role in multiple cellular processes including immune response and cell cycle progression.

## Graphical Abstract

**Figure:**
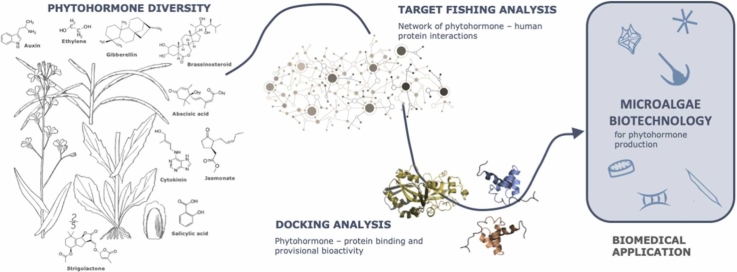

## 1 Introduction

Photosynthetic organisms synthetize a plethora of secondary metabolites; most of them are bioactive molecules improving their attractiveness as natural resource for human health protection [1], [2], [3]. To reach the human health industrial market – e.g., nutraceuticals, food complements or cosmeceuticals - the productive process of the natural bioactive compound needs to be economically and environmentally sustainable. In this regard, microalgae are greatly attractive compared to plants [4], [5], thanks to their overall uniqueness and the possibility to be used as cell factory [5]. Indeed, research efforts to implement microalgae as human health benefit-resource are exponentially increasing [5], [6], [7]. Phytohormones (PHs) are among the less investigated secondary metabolites in microalgae and therefore require scientific efforts to unravel their potential biotechnological interests [8], [9]. Phytohormones, a group of small bioactive secondary metabolites present in all photosynthetic organisms [10] are conserved polyfunctional molecules from different families, including auxins (AUXs), cytokinins (CKs), gibberellins (GAs), jasmonic acid (JA), abscisic acid (ABA), salicylic acid (SA), strigolactones (SLs) and brassinosteroids (BRs). They contribute into many relevant plant functions, such as cell division, homeostasis, growth, modulation of plant-microbe interactions, defense against pathogens, phototropism or memory [10]. Previous studies report PHs synthesis in microalgae [8], in which they endow roles of regulation of fundamental processes as growth and homeostasis, as well as for extracellular signaling [8], [11]. Beside their crucial roles in photosynthetic organisms, PHs might also benefit human or animal health [12], [13] and the exploration of their bioactivity is gaining of interest [13]. However, there is a great disparity on the knowledge among the huge number of PH compounds. Some families were more investigated than others. This is the case of abscisic acid involved in glycemic control [14], [15] and of salicylic acid [16], [17]. Some auxins displayed anti-tumor function [18]. Some cytokinins had reveal different roles, such as neuroprotective, antioxidant, anticancer, anti-inflammatory [19], [13] while some brassinosteroids displayed chemopreventive, antiangiogenic, antiviral, or anti-inflammatory activities [20], [21]. Some bioactivities such as the modulation of gut-microbiota interactions in human were commonly reported among auxins, cytokinins and abscisic acid [12], [22]. Conversely, many compounds from these families and from other families (e.g., jasmonates, gibberellins, strigolactones) were still little investigated. Since the plethora of PH compounds, with for instance more than 70 species of brassinosteroids, or 250 members of gibberellins [3], a low cost and fast screening strategy should be established to select some compounds to be further explored through in vitro assays.

Our study addresses the high diversity-related gap of knowledge on PH bioactivity proposing a large bioprospecting on the interactions between PH compounds and human proteins or cell receptors involved in metabolism, cell growth or division and immune system functioning. This was done through an *in silico* analysis, i.e. a target fishing approach. The most five significant interactions for each PH compound were then discussed both in term of human protein roles and PH family. As a second step, an *in silico* modeling through molecular docking analysis was performed with the aim to deeply investigate the ligand-receptor interactions when the latter were specific, i.e. unique for the PH compound. This approach allowed to screen fifty-three PH compounds, highlighting the potential human health benefit interests of some of them, inferring their activities on human metabolism and health protection.

## 2 Materials and methods

### 2.1 Target fishing and network analysis

Fifty-three naturally occurring phytohormones (PHs) were selected in order to cover a large PH chemical diversity, including 22 cytokinins (CKs), 11 auxins (AUXs), 6 gibberellins (GAs), 6 strigolactones (SLs), 4 jasmonates (JAs), 2 brassinosteroids (BRs), abscisic acid (ABA) and salicylic acid (SA) (Table 1). An introductory target fishing approach was carried out to explore the interactions between these 53 PHs and proteins involved in human metabolism, cell growth or division and immune system functioning by using ACID online server [23]. From the outputs of this target fishing exploration, we selected the five best interactions between PHs and human protein based on the highest scores of interaction binding energy. These results were then included in a network analysis generated with GraphCommons online software [24].

**Table 1:** List of the fifty-three phytohormones investigated in this study.

**Class**	**Compound name**	**Abbreviations**	**CID**
**abscisic acid**	(+)-abscisic acid	ABA	5280896
**auxins**	2-oxindole-3-acetic acid	oxIAA	3080590
	2-phenylacetic acid	PAA	10011961
	4-chloroindole-3-acetic acid	4-CI-IAA	100413
	indole-3-acetaldoxime	IAOx	5371769
	indole-3-acetamide	IAM	397
	indole-3-acetic acid	IAA	802
	indole-3-acetonitrile	IAN	351795
	indole-3-butyric acid	IBA	8617
	indole-3-carboxylic acid	ICA	69867
	indole-3-propionic acid	IPA	3744
	indole-3-pyruvic acid	IPA	803
**brassinosteroids**	brassinolide	BL	115196
	castasterone	CS	133534
**cytokinins**	6-benzylaminopurine	6-BAP	62389
	cis-zeatin	cZ	688597
	cis-zeatin riboside	cZR	13935024
	cis-zeatin riboside-5′-monophosphate	cZRMP	23724752
	cis-zeatin-9-glucoside	cZ9G	101921807
	cis-zeatin-O-glucoside	ZOG	5280589
	dihydrozeatin	DHZ	32021
	dihydrozeatin riboside	DHZR	10522005
	dihydrozeatin riboside-5′-monophosphate	DHZMP	72989203
	dihydrozeatin-9-glucoside	DHZ9G	73002011
	dihydrozeatin-O-glucoside	DHZOG	23724755
	kinetin	K	3830
	N6-(D2-isopentenyl)adenine	iP	92180
	N6-(D2-isopentenyl)adenosine	iPR	24405
	N6- (D2-isopentenyl)adenosine-5′-monophosphate	iPRMP	10180201
	N6-(D2- isopentenyl)adenine-9-glucoside	iP9G	25200472
	trans-zeatin	tZ	449093
	trans-zeatin riboside	tZR	6440982
	trans-zeatin riboside- 5′-monophosphate	tZRMP	11561034
	trans-zeatin riboside-O-glucoside	tZROG	131751341
	trans-zeatin-9-glucoside	tZ9G	9842892
	trans-zeatin-O-glucoside	tZOG	5461146
**gibberellins**	gibberellin A1	GA1	5280379
	gibberellin A3	GA3	6466
	gibberellin A4	GA4	92109
	gibberellin A5	GA5	443464
	gibberellin A6	GA6	443449
	gibberellin A7	GA7	92782
**jasmonates**	jasmonic acid	JA	5281166
	12-oxo-phytodienoic acid	OPDA	5280411
	dinor-12-oxo-phytodienoic acid	dinor-OPDA	644074
	methyl jasmonate	MeJA	5281929
**salicylic acid**	salicylic acid	SA	338
**strigolactones**	(+)− 5-deoxystrigol		5281396
	(+)-orobanchol		15102669
	(+)-orobanchyl acetate		10665247
	(+)-strigol		24796587
	(+)-strigyl acetate		15102684
	sorgolactone		5281395

### 2.2 Molecular docking analysis

The interactions between PHs and human proteins resulting as non-redundant, i.e., specific within a PH class were further subjected to *in silico* modeling through molecular docking analysis. The 3D coordinates of crystal structures of the selected targets were recovered from Protein Data Bank - PDB. The protein structure was then used as the receptor model and optimized by UCSF (University of California San Francisco) Chimera 1.16 software for removal of all heteroatoms and water molecules included in PDB files [25]. Further polar hydrogen atoms were added to proteins to make the receptor molecules suitable for docking [26]. The same procedure was applied for the PHs (ligands). Gasteiger charges were added and a maximum of six numbers of active torsions were given to the lead compounds [27]. Once the ligand and receptor preparation done, the selected proteins were finally docked using AutoDock Vina 4.2.6 [28].

## 3 Results and discussion

**Fig. 1:**
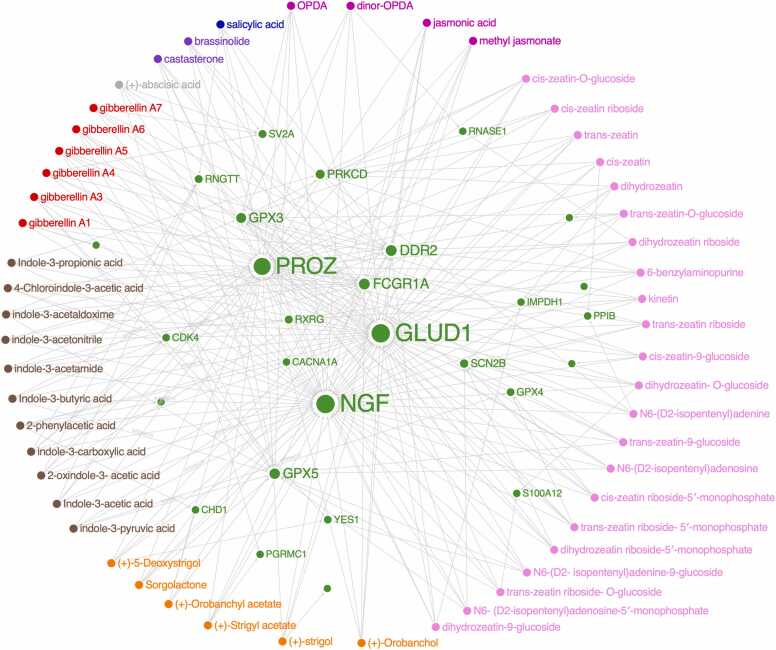
Network of phytohormones’ interactions with human target proteins (green) as retrieved from target fishing analysis. The size of the target-dot is determined by the number of interactions. Colors indicate the different classes of hormones: pink for CKs, fuchsia for JAs, blue for SA, purple for BRs, gray for ABA, red for GAs, brown for AUXs, or orange for SLs. See Table 1 and supplementary Table S1 for phytohormones and human targets properties.

**Fig. 2:**
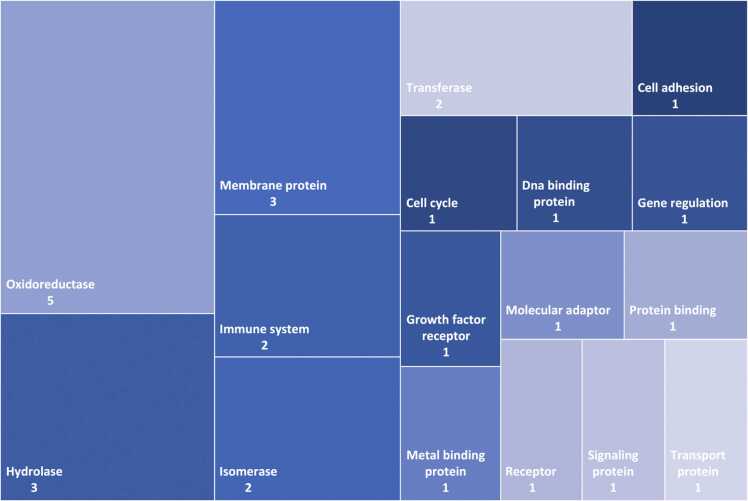
Classification of the human targets identified by the target fishing analysis. Size of rectangles is proportional to the number of targets within the class.

Significant interactions between the fifty-three PHs (Table 1) with twenty-eight human targets were reported (Fig. 1; Supplementary Table S1). The retrieved human targets were involved in immunomodulation (52 PHs), oxidative stress response (50 PHs), signal transduction (22 PHs) or cytoskeletal structural role (10 PHs) (Fig. 2).

### 3.1 Overview of the interactions of the phytohormones with human targets

While some interactions were ligand-receptor specific (see the following sections), most of them were shared among many PHs and receptors. This was the case of the three targets mitochondrial glutamate dehydrogenase (GLUD1), nerve growth factor (NGF) and vitamin K-dependent protein Z (PROZ). GLUD1 and NGF proteins interacted with 51 PHs, with at least one compound within each class of PHs. Only the 6-benzylaminopurine (cytokinin) and the 2-oxindole-3-acetic acid (auxin) did not present binding affinity with GLUD1, while the cytokinin trans-zeatin-O-glucoside and the auxin indole-3-acetaldoxime could not interact with NGF. PROZ interacted with 45 PHs while it did not present binding affinity with abscisic acid, 2-phenylacetic acid (auxin), the gibberellins A1, A3, A5, A7, the brassinosteroid brassinolide and the cytokinin N6-(D2-isopentenyl) adenine-9-glucoside.

The affinity between a huge number of PHs and the three receptors might be explained by the fact these proteins are involved in the human gut functioning i.e., relied to gut-microbiota interactions [12], [13]. GLUD1 expression is involved in amino-acid metabolism, and especially the glutamine and glutamate-associated pathways [29], while a significant GLUD1 increase was observed in germ-free mice [30]. NGF promotes innervation and proliferation in gastric epithelium and is involved in gastric tumor development [31]. The vitamin K dependent protein Z (PROZ) synthesized in the liver and then secreted into the plasma [32] was recently associated with Type 2 diabetes mellitus (T2DM). A decreased PROZ level was observed in patients with prediabetes or manifesting T2DM [33].

While most of the PH compounds presented recurrent interactions with human targets, eight PHs from the auxin, cytokinin, gibberellin, or strigolactone families presented specific interactions with one receptor (Table 2). To deeply investigate these compounds, they were thus targeted with molecular docking analysis (see 3.5, 3.6, 3.7, 3.8 below).

**Table 2:** Identities and scores of the specific interactions revealed between phytohormones and human targets. PDB ID: Protein data bank identifier; RMSD: root-mean-square deviation; PubChem CID: Chemical Identifier.

**Class**	**Compound**	**CID**	**Target**	**Full name**	**PDB ID**	**RMSD**	**H-bonds**	**E binding kcal mol**^**−1**^**(ACID)**	**E binding kcal mol**^**−1**^**(chimera)**
Auxin	2-phenylacetic acid	999	DLG4	Disks large homolog 4	3K82	0.0	2	-5.47	-5.2
Cytokinin adenine-type	cis-zeatin	688597	PPIB	Peptidyl-prolyl cis-trans isomerase B	3ICI	0.0	1	-5.59	-5.6
	dihydrozeatin	32021	IMPDH1	Inosine-5′-monophosphate dehydrogenase 1	1JCN	0.0	1	-5.57	-5.5
	kinetin	3830	CAD	CAD protein	4C6E	0.0	1	-5.58	-7.0
	N6-(D2-isopentenyl)adenine	92180	PPIB	Peptidyl-prolyl cis-trans isomerase B	3ICI	0.0	0	-5.59	-5.9
	trans-zeatin-O-glucoside	5461146	SPSB2	SPRY domain-containing SOCS box protein 2	3EMW	0.0	3	-5.23	-6.3
Gibberellin	gibberellin A1	5280379	CLCNKA	Chloride channel protein ClC-Ka	2PFI	0.0	2	-6.24	-6.9
Strigolactones	(+)-strigol	5281396	PPIC	Peptidyl-prolyl cis-trans isomerase C	2ESL	0.0	1	-6.42	-8.5

### 3.2 Jasmonates

Among the other redundant interactions between PHs and human targets, the four compounds from the class jasmonates displayed a number of connections involving targets associated with tumor progression (CACNA1A), oxidative stress management (GPX3, GPX5) and apoptosis (DDR2) (Fig. 1, Supplementary Table S1). This result agrees with previous studies [34] reporting that jasmonates presented in vitro ability to initiate cancer cell death by activating several mechanisms such as ATP depletion, MAPK induction, and ROS production.

### 3.3 Brassinosteroids

The two brassinosteroids, castasterone and brassinolide, interacted with proteins with function involved in apoptosis (DDR2), signaling (SV2A), oxidative stress management (GPX5) or immune response (FCGR1A) (Fig. 1, Supplementary Table S1). Indeed, anticancer activities of brassinosteroids had been reported [35]. For instance, brassinosteroids inhibited cells proliferation in several tumor cell lines inducing apoptosis [36], [37] and induced the suppression of endothelial cells migration and angiogenesis [37].

### 3.4 Abscissic acid

Among the protein interactions predicted for abscissic acid, binding affinity with synaptic vesicle glycoprotein 2A (SV2A) and membrane-associated progesterone receptor component 1 (PGRMC1) was highlighted (Fig. 1, Supplementary Table S1). SV2A is involved in calcium-stimulated exocytosis and priming of synaptic vesicles [38], and its expression increases in response to pharmacological treatment in glioblastoma [38]. Indeed, ABA is noteworthy to induce autophagy mediated by MAPK/JNK signaling in mice glioblastoma cells [39]. PGRMC1 is a protein with multiple function which binds progesterone and pharmaceutical compounds and thus becomes an attractive target for anticancer activities [40].

### 3.5 Salicylic acid

Salicylic acid shared with other PHs binding affinity with PROZ, GLUD1, NGF, DDR2 and the mRNA-capping enzyme (RNGTT) (Fig. 1, Supplementary Table S1), revealing its potential other bioactivity beyond its use as nonsteroidal anti-inflammatory drug [41].

### 3.6 Auxins

Auxins interacted primarily with proteins involved in oxidative stress management (GLUD1, GPX3, GPX4, GPX5) and immune system response (FCGR1A, NGF). Moreover, receptors with function in DNA binding (CHD1), cell cycle progression (CDK4), growth factor receptor (RXRG), hydrolase (PROZ), membrane protein (CACNA1A), binding protein (PRKCD), kinase (DDR2) and transferase (RNGTT) were also targeted by some auxins (Fig. 1, Supplementary Table S1).

**Fig. 3:**
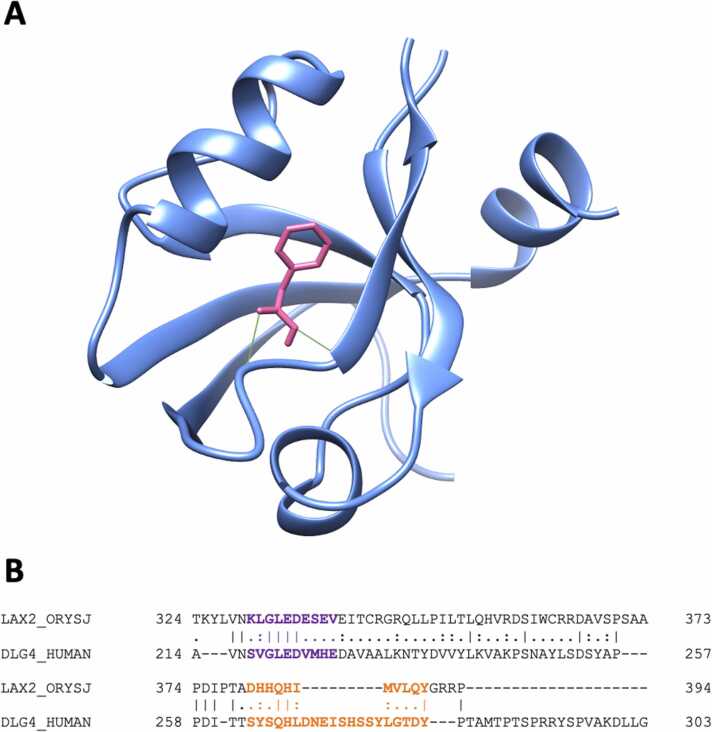
(**a**) Molecular docking of the auxin 2-phenylacetic acid (PAA) to Disks large homolog 4 (DLG4) protein; (**b**) the PDZ binding motif (purple) and the amino acids required for its interaction with OsIAA3 and OsIAA17 in *O. sativa* according to [43] (orange); identical positions (|), conserved substitutions (:) and semi-conserved substitutions (·) are marked below the sequences.

The target human disks large homolog 4 was specifically bound by the auxin phenylacetic acid (PAA; Table 2; Fig. 3(**a**)). DLG4 is involved in the microglial inflammatory response [42] and in postsynaptic scaffolding playing a critical role in synaptogenesis and related neurodegenerative diseases [43]. The DLG4 protein contains three domains, so-called PDZ, at the N-terminus and a SH3–GK tandem at the C-terminus [44]. PDZ domains-containing proteins are generally involved in signaling at cellular membranes, with PDZ domains play a key role in anchoring membrane proteins to cytoskeletal components [45].

It is known that in plants auxin signaling may act directly on key protein domains, such as PDZ and OsIAA protein [46] and that the human protein DLG4 displays homology with both binding domains PDZ and OsIAA from the *Oryza sativa* protein Gnp4/LAX2 (GenBank: KY673700.1; Fig. 3(**b**)).

### 3.7 Cytokinins

Among cytokinins, the targets mainly belonged to oxidative stress management (GLUD1, GPX3, GPX4, GPX5) or to immune system response (FCGR1A, NGF). Other receptors bound by cytokinins were affiliated to growth factor (RXRG), hydrolase (RNASE1, PROZ), membrane protein (SCN2B), binding protein (PRKCD), kinase (DDR2), and transferase (YES1) (Fig. 1, Supplementary Table S1).

**Fig. 4:**
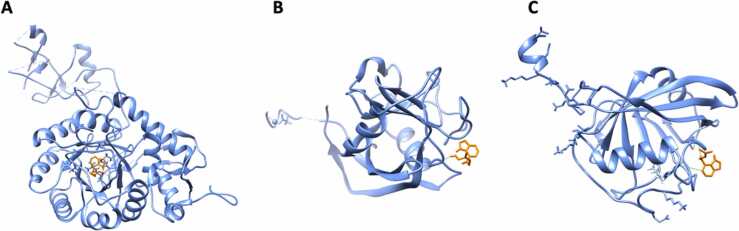
Molecular docking of: (**a**) dihydrozeatin (DHZ) to Inosine-5′-monophosphate dehydrogenase 1 (IMPDH1); (**b** and **c**) Cis-zeatin and N6-(D2-isopentenyl)-adenine to Peptidyl-prolyl cis-trans isomerase B (PPIB) protein, respectively.

Over the twenty-two CKs used for target fishing, five displayed specific interactions with only one human target (Table 2). Dihydrozeatin (DHZ) displayed binding affinity for Inosine-5′-monophosphate dehydrogenase 1 (IMPDH1) protein (Table 2; Fig. 4 (**a**)). The binding was due to the formation of a hydrogen bond between DHZ and Gly-326 (Fig. 4 (**a**)), situated into the Inosine-5′-monophosphate dehydrogenase / Guanosine 5′-monophosphate oxidoreductase conserved site (IPR015875: IMP_DH/GMP_Rdtase_CS, 321 – 333).

IMPDH1 plays an important role in the regulation of cell growth and proliferation [47] catalyzing the conversion of inosine 5′-phosphate (IMP) to xanthosine 5′-phosphate (XMP). Although IMPDH1 is ubiquitously expressed, it plays a critical role in cyclic nucleoside metabolism within photoreceptors [48]. Indeed, mutations in IMPDH1 are tightly linked with *retinitis pigmentosa,* a rare eye genetically-determined disease [48]. Conversely, IMPDH1 upregulation determines the progression of acute lymphoblastic leukemia (ALL) tumors, in modulating purine biosynthesis [49]. This key-protein is the purpose of several drugs with antitumor or antiviral functions e.g., tiazofurin and ribavirin, respectively [50], [51]. Also, some drugs e.g., mizoribine or mycophenolic acid act as immunosuppressive ligands to inhibit IMPDH1 [52]. Comparative analysis of the energy score of the binding between IMPDH1 and Dihydrozeatin or these four drugs revealed a great potential of DHZ to establish stable binding with the receptor (Supplementary Table S2).

The two cytokinins cis-zeatin (cZ) and N6-(D2-isopentenyl)-adenine (iP) bound the peptidyl-prolyl cis-trans isomerase B (PPIB) protein (Table 2; Fig. 4 (**b, c**)). PPIB is a cyclophilin that catalyzes the cis-trans isomerization of proline imidic peptide bonds in oligopeptide being therefore involved in protein folding [53]. In general, cyclophilins play a role in several intracellular processes such as oxidative stress, mitochondrial dysfunction, cell migration and apoptosis, with consequences in the development of cardiovascular diseases, neurodegeneration, cancer or viral infection [54], [55]. PPIB promotes cell proliferation and angiogenesis by regulating the signal transduction and activation of the transcription-3 pathway in non-small-cell lung cancer [56] while its overexpression enhanced HIV-1 infection by increasing the nuclear import of viral DNA [57], [58]. The docked ligands were harbored in different sites, close to the PPIase conserved site (IPR020892, Cyclophilin-type_PPIase_CS: 60–77). Cis-zeatin formed an H-bond with Ser-59, while N6-(D2-isopentenyl)-adenine formed two H-bonds with Asn-61 and Phe-57, respectively. PPI1 domain is the only responsible for the protein activity in both human and plant cyclophilins [58], [59] suggesting that adenine-type cytokinins recognize plant protein domains, as PPI, homology in humans.

**Fig. 5:**
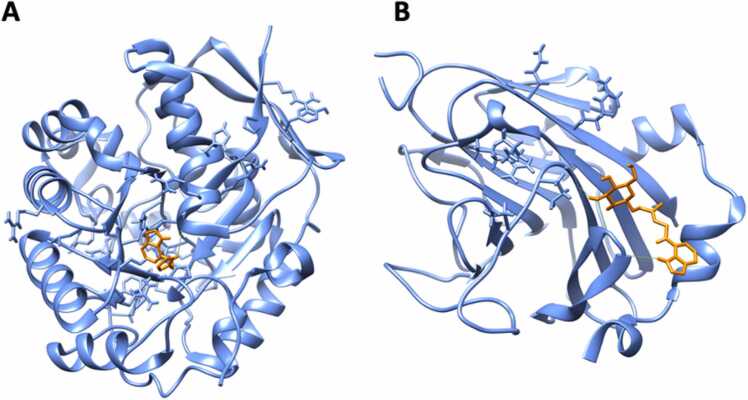
Molecular docking of: (**a**) trans-zeatin-O-glucoside (tZOG) to SOCS box protein 2 (SPSB2) (**b**) Kinetin to carbamoyl-phosphate synthetase 2, aspartate transcarbamylase, and dihydroorotase protein (CAD protein).

Trans-zeatin-O-glucoside (tZOG) did only interact with SOCS box protein 2 (SPSB2) (Table 2; Fig. 5 (**a**)). The SPRY domain (IPR003877: 86 – 220) of SPSB2 hosted the interaction with tZOG, that forms three H-bonds in the C-terminus of the SPSB2 SPRY domain, binding Arg-166, Gly-183 and Thr-185. SPSB2 does recruit E3 ubiquitin ligase complex to polyubiquitinate iNOS by interacting with its N-terminus, thus determining proteasome activation and subsequent increase in NO production [60]. Also, SPSB2 can exert an antiviral function by binding structural protein E1 and nonstructural protein 5A (NS5A) from *Hepatitis C Virus* (HCV), inhibiting its replication [61].

These findings suggest that tZOG may act in neurodegenerative diseases [62] and in antiviral response [63], through its interaction with SPSB2.

Kinetin (K) showed binding affinity for Dihydroorotase protein (CAD protein, Table 2; Fig. 5 (**b**)). The binding between CAD and kinetin involved a H-bond formed by K with Ile-280, with a high energy binding affinity (−7.0 kcal mol^−1^). CAD protein is a trifunctional multi-domain enzyme involved in the first three steps of pyrimidine biosynthesis [64]. Kinetin was already described for its bioactivity potential, for instance its ability to delay the onset of ageing features in human fibroblasts [65], [66] and its activity to rescue mRNA splicing defects in humans [67], [68]. Also, kinetin displayed protection against oxidative damage and enhanced cell viability in cultured astrocytes [69], while might be a catabolite linked to oxidative damage process in human [70], [71].

### 3.8 Gibberellins

Gibberellins were mainly interacting with proteins involved in the immune system response (FCGR1A, NGF) or in oxidative stress management (GLUD1, GPX5). Nonetheless, binding to proteins with function in signaling (SV2A), or as hydrolase (PROZ), kinase (DDR2), and transferase (YES1) were displayed (Fig. 1, Supplementary Table S1).

**Fig. 6:**
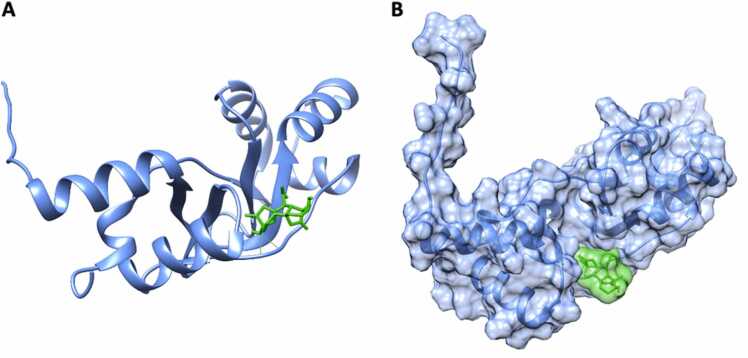
Binding affinity between Gibberellin A1 (GA1) and chloride channel protein ClC-Ka (CLCNKA): (**a**) cartoon; (**b**) surface energy plot.

Specific interaction was found between the gibberellin A1 (GA1) wand the human chloride channel protein ClC-Ka (CLCNKA) (Table 2; Fig. 6 (**a, b**)). Binding took place through two H-bonds, both occurring with Thr-632 (Interpro domain: cd04591). CLCNKA is a voltage-gated chloride channel involved in the regulation of cell volume, membrane potential stabilization, signal transduction and transepithelial transport [72], especially in kidneys [73]. Indeed, CLCNKA insufficiency induces salt loss in the urine, and disrupts the normal balance of ions in the body in relation with Bartter syndrome and hypertension [74].

### 3.9 Strigolactones

As for Gibberellins, strigolactones displayed binding with proteins involved in immune system response (FCGR1A, NGF) and oxidative stress management (GLUD1, GPX5). Moreover, some target receptors with function in DNA binding (CHD1), hydrolase (PROZ), kinase (DDR2) and progesterone receptor (PGRMC1, Fig. 1, Supplementary Table S1).

**Fig. 7:**
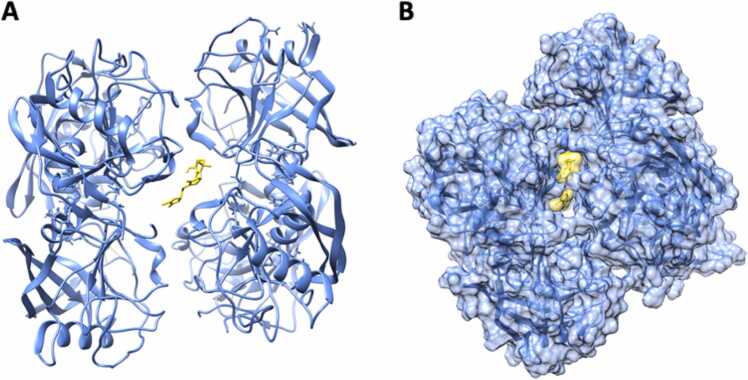
Binding affinity between (+)-strigol and peptidyl-prolyl cis-trans isomerase C (PPIC): (**a**) cartoon; (**b**) surface energy plot.

Among the six strigolactones, only (+)-strigol displayed a specific binding affinity, i.e. with the Peptidyl-prolyl cis-trans isomerase C (PPIC) (Table 2; Fig. 7 (**a**)). PPIC tetramer harbors the docked ligand in the pocket site, supported by a substantial binding energy score (−8.5 kcal mol^−1^) resulting by solely hydrophobic interaction (Fig. 7 (**b**)). PPIC is a cyclophilin which plays different roles in human health [54], [55], [75]. Antiviral and anticancer activities of natural or synthetic strigolactones have been documented [76], [77]. Also, the activity of stereo-defined strigolactones has been acknowledged for high cancer cell specificity in humans [77], [78]. Recently, PPIC has been shown as directly involved in B lymphocytes and invariant Natural Killer T-cells (iNKT) differentiation [79], while high levels of PPIC were correlated to coronary artery diseases [80]. The biological activity of strigolactones toward tumor treatment or blood pressure control in humans has been attributed to the α-methylene-γ-lactone group (αMγL), an oxygen-containing ring structure with a carbonyl function [81].

## 4 Conclusions

In a bioprospecting perspective, our *in silico* study aimed to screen the predictable interactions between 53 PHs and human receptors, first step then to infer their potential bioactivities. Among the 28 retrieved human targets mainly involved in immune response, oxidative stress or cell cycle progression, most of them interacted with many PHs presenting redundant predicted binding affinities. Our findings support the hypothesis that phytohormone compounds might have antimicrobial, antiviral or antitumor functions. When bioactivity results are available, our study corroborated the information e.g., the anticancer ability of some brassinosteroids, or jasmonates as well as the protective capacity against oxidative damage of kinetin. Conversely, eight PHs targeted specific human receptors: five cytokinins, one auxin, one strigolactone and one gibberellin. Some these compounds are greatly worth of interest e.g., the two cytokinins, dihydrozeatin (DHZ) and trans-zeatin-o-glucoside (tZOG), which strongly and specifically interacted with IMPDH1 and SPSB2 receptors, respectively. The former (IMPDH1) is a known target in cancer therapy that might made DHZ as a potential chemoprotective compound, while the latter (SPSB2) has an antiviral role, leading tZOG to hypothetically be an antiviral compound.

This *in silico* assessment of PHs-human protein interactions paves the way to select compounds of potential interest to explore their in vitro and/or in vivo bioactivity. Bioactivity of some PHs might further enrich the interests for microalgae as a resource for the market of human health benefits products, together with carotenoids, polyphenols or vitamins [82], [5], [6].

## Funding

This research was funded by 10.13039/501100010685Stazione Zoologica Anton Dohrn and by “Antitumor Drugs and Vaccines from the Sea (ADViSE)” project (PG/2018/0494374). Angelo del Mondo was supported by a post-doctoral fellowship funded by “Antitumor Drugs and Vaccines from the Sea (ADViSE)” project (PG/2018/0494374). Luigi Pistelli is supported by a PhD fellowship co-funded by “Antitumor Drugs and Vaccines from the Sea (ADViSE)” project PG/2018/0494374) and by the 10.13039/501100010685Stazione Zoologica Anton Dohrn (PhD Program XXI cycle, Open University, Milton Keynes, UK).

## CRediT authorship contribution statement

**ADM**: Methodology, Formal analysis, Visualization, Writing – original draft, Writing - review & editing. **AV:** Formal analysis, Visualization, Writing - review & editing. **LP:** Formal analysis, Visualization, Writing - review & editing**. CB**: Conceptualization, Writing - review & editing, Project administration. **CS**: Conceptualization, Writing - review & editing, Project administration.

## Declaration of Competing Interest

The authors declare that they have no known competing financial interests or personal relationships that could have appeared to influence the work reported in this paper.
