# Supplementary material for: On the human health benefits of microalgal phytohormones: An explorative in silico analysis
Source: Comput Struct Biotechnol J. 2023 Jan 25;21:1092–101. doi: 10.1016/j.csbj.2023.01.032 (PMC9900276; doi:10.1016/j.csbj.2023.01.032)
Supplement: Supplementary file 1 — Supplementary material [file mmc1.docx]

**Supplementary Table S1**

Results of the target fishing analysis between the 53 phytohormones and human targets. For each PH, the best five targets in term of binding energy score are reported

| **class** | **Phytohormone compounds** | **Human Targets** | **Target name** | **Classification** | **Uniprot ID** | **PDB code** |
| --- | --- | --- | --- | --- | --- | --- |
|  |  | GLUD1 | Glutamate dehydrogenase 1, mitochondrial | Oxidoreductase | P00367 | 1L1F |
|  |  | DDR2 | Discoidin domain-containing receptor 2 | Receptor | Q16832 | 2WUH |
|  | (+)-abscisic acid | NGF | Beta-nerve growth factor | Immune system | P01138 | 4ZBN |
| Abscisic acid |  | PGRMC1 | Membrane-associated progesterone receptor component 1 | Membrane protein | O00264 | 4X8Y |
|  |  | SV2A | Synaptic vesicle glycoprotein 2A | Signaling protein | Q7L0J3 | 4V11 |
|  |  | PROZ | Vitamin K-dependent protein Z | Hydrolase inhibitor / hydrolase | P22891 | 3F1S |
|  |  | NGF |  |  |  |  |
|  | 2-oxindole-3-acetic acid | FCGR1A | High affinity immunoglobulin gamma Fc receptor I | Immune system | P12314 | 3RJD |
|  |  | RNGTT | mRNA-capping enzyme | Transferase | O60942 | 2C46 |
|  |  | CDK4 | Cyclin-dependent kinase 4 | Cell cycle | P11802 | 2W96 |
|  |  | GLUD1 |  | | | |
| Auxins |  | NGF |  |  |  |  |
|  |  | DLG4 | Disks large homolog 4 | Cell adhesion | P78352 | 3K82 |
|  | 2-phenylacetic acid | CACNA1A | Voltage-dependent P/Q-type calcium channel subunit alpha-1A | Membrane protein | O00555 | 3BXK |
|  |  | CHD1 | Chromodomain-helicase-DNA-binding protein 1 | DNA binding protein | O14646 | 4O42 |
|  |  | GLUD1 |  | | | |
|  |  | NGF |  |  |  |  |
|  | 4-Chloroindole-3-acetic acid | PROZ |  |  |  |  |
|  |  | FCGR1A |  |  |  |  |
|  |  | CDK4 |  |  |  |  |
|  |  | GLUD1 |  |  |  |  |
|  |  | GPX5 | Epididymal secretory glutathione peroxidase | Oxidoreductase | O75715 | 2I3Y |
| Auxins | indole-3-acetaldoxime | PROZ |  | | | |
|  |  | FCGR1A |  |  |  |  |
|  |  | GPX3 | Glutathione peroxidase 3 | Oxidoreductase | P22352 | 2R37 |
|  |  | GLUD1 |  | | | |
|  |  | PROZ |  |  |  |  |
|  |  | NGF |  |  |  |  |
|  | indole-3-acetamide | FCGR1A |  |  |  |  |
|  |  | RXRG | Retinoic acid receptor RXR-gamma | Growth factor receptor | P48443 | 2GL8 |
|  |  | GLUD1 |  | | | |
|  |  | PROZ |  |  |  |  |
|  |  | NGF |  |  |  |  |
|  | indole-3-acetic acid | RNGTT |  |  |  |  |
|  |  | PRKCD | Protein kinase C delta type | Protein binding | Q05655 | 1YRK |
|  |  | GLUD1 |  | | | |
|  |  | PROZ |  |  |  |  |
|  |  | NGF |  |  |  |  |
|  | indole-3-acetonitrile | DDR2 |  |  |  |  |
|  |  | RXRG |  |  |  |  |
|  |  | GLUD1 |  |  |  |  |
|  |  | PROZ |  |  |  |  |
|  | indole-3-butyric acid | NGF |  |  |  |  |
|  |  | GPX5 |  |  |  |  |
|  |  | DDR2 |  |  |  |  |
|  |  | GLUD1 |  |  |  |  |
|  |  | PROZ |  |  |  |  |
|  | indole-3-carboxylic acid | NGF |  |  |  |  |
|  |  | RNGTT |  |  |  |  |
|  |  | CDK4 |  |  |  |  |
| Auxins |  | GLUD1 |  |  |  |  |
|  |  | NGF |  |  |  |  |
|  | indole-3-propionic acid | FCGR1A |  |  |  |  |
|  |  | PRKCD |  |  |  |  |
|  |  | PROZ |  |  |  |  |
|  |  | GLUD1 |  |  |  |  |
|  |  | PROZ |  |  |  |  |
|  | indole-3-pyruvic acid | NGF |  |  |  |  |
|  |  | GPX4 | Phospholipid hydroperoxide glutathione peroxidase | Oxidoreductase | P36969 | 5H5Q |
|  |  | GPX5 |  | | |  |
|  |  | GLUD1 |  | | | |
|  |  | NGF |  |  |  |  |
|  | brassinolide | DDR2 |  |  |  |  |
|  |  | SV2A |  |  |  |  |
|  |  | GPX5 |  |  |  |  |
|  |  | GLUD1 |  |  |  |  |
|  |  | NGF |  |  |  |  |
| Brassinosteroids |  | FCGR1A |  |  |  |  |
|  | castasterone | PROZ |  |  |  |  |
|  |  | GPX5 |  |  |  |  |
|  |  | PROZ |  | | | |
|  |  | NGF |  |  |  |  |
|  | 6-benzylaminopurine | RXRG |  |  |  |  |
|  |  | DDR2 |  |  |  |  |
|  |  | PRKCD |  |  |  |  |
|  |  | GLUD1 |  |  |  |  |
|  |  | PROZ |  |  |  |  |
|  | cis-zeatin | NGF |  |  |  |  |
|  |  | PRKCD |  |  |  |  |
|  |  | PPIB | Peptidyl-prolyl cis-trans isomerase B | Isomerase | P23284 | 3ICI |
| Cytokinins |  | GLUD1 |  | | | |
|  |  | PROZ |  |  |  |  |
|  |  | NGF |  |  |  |  |
|  | cis-zeatin riboside | GPX3 |  |  |  |  |
|  |  | PRKCD |  |  |  |  |
|  | cis-zeatin riboside-5′-monophosphate | GLUD1 |  |  |  |  |
|  |  | PROZ |  |  |  |  |
|  |  | NGF |  |  |  |  |
|  |  | SCN2B | Sodium channel subunit beta-2 | Membrane protein | O60939 | 5FEB |
|  |  | GPX4 |  | | | |
|  |  | GLUD1 |  |  |  |  |
|  |  | PROZ |  |  |  |  |
|  |  | NGF |  |  |  |  |
|  | cis-zeatin-9-glucoside | GPX3 |  |  |  |  |
|  |  | S100A13 | Protein S100-A13 | Gene regulation | Q99584 | 3N4M |
|  |  | GLUD1 |  | | | |
|  |  | NGF |  |  |  |  |
|  |  | PROZ |  |  |  |  |
|  | cis-zeatin-O-glucoside | GPX3 |  |  |  |  |
|  |  | PRKCD |  |  |  |  |
|  |  | GLUD1 |  |  |  |  |
|  |  | NGF |  |  |  |  |
|  |  | PROZ |  |  |  |  |
|  | dihydrozeatin | IMPDH1 | Inosine-5'-monophosphate dehydrogenase 1 | Oxidoreductase | P20839 | 1JCN |
| Cytokinins |  | PRKCD |  | | | |
|  |  | GLUD1 |  |  |  |  |
|  |  | NGF |  |  |  |  |
|  | dihydrozeatin riboside | PROZ |  |  |  |  |
|  |  | RNASE1 |  |  |  |  |
|  |  | DDR2 |  |  |  |  |
|  |  | GLUD1 |  |  |  |  |
|  |  | PROZ |  |  |  |  |
|  | dihydrozeatin riboside-5′-monophosphate | NGF |  |  |  |  |
|  |  | SCN2B |  |  |  |  |
|  |  | GPX3 |  |  |  |  |
|  |  | GLUD1 |  |  |  |  |
|  |  | PROZ |  |  |  |  |
|  |  | NGF |  |  |  |  |
|  | dihydrozeatin-9-glucoside | IMPDH1 |  |  |  |  |
|  |  | DDR2 |  |  |  |  |
|  |  | GLUD1 |  |  |  |  |
|  |  | PROZ |  |  |  |  |
|  | dihydrozeatin-O-glucoside | NGF |  |  |  |  |
|  |  | S100A12 | Protein S100-A12 | Metal binding protein | P80511 | 2WCB |
|  |  | SCN2B |  | | | |
|  |  | GLUD1 |  |  |  |  |
|  |  | PROZ |  |  |  |  |
|  | kinetin | NGF |  |  |  |  |
|  |  | RXRG |  |  |  |  |
|  |  | CAD | CAD protein | Hydrolase | P27708 | 4C6E |
| Cytokinins |  | GLUD1 |  | | | |
|  |  | PROZ |  |  |  |  |
|  | N6-(D2-isopentenyl)adenine | NGF |  |  |  |  |
|  |  | PRKCD |  |  |  |  |
|  |  | PPIB |  |  |  |  |
|  |  | GLUD1 |  |  |  |  |
|  |  | NGF |  |  |  |  |
|  | N6-(D2-isopentenyl) adenosine | PROZ |  |  |  |  |
|  |  | GPX3 |  |  |  |  |
|  |  | GPX5 |  |  |  |  |
|  |  | GLUD1 |  |  |  |  |
|  |  | PROZ |  |  |  |  |
|  | N6- (D2-isopentenyl) adenosine-5′-monophosphate | NGF |  |  |  |  |
|  |  | SCN2B |  |  |  |  |
|  |  | GPX5 |  |  |  |  |
|  |  | GLUD1 |  |  |  |  |
|  |  | NGF |  |  |  |  |
|  | N6-(D2-isopentenyl) adenine-9-glucoside | GPX3 |  |  |  |  |
|  |  | GPX5 |  |  |  |  |
|  |  | YES1 | Tyrosine-protein kinase Yes | Transferase | P07947 | 2HDA |
|  |  | GLUD1 |  | | | |
|  |  | PROZ |  |  |  |  |
|  |  | NGF |  |  |  |  |
|  | trans-zeatin | PRKCD |  |  |  |  |
|  |  | FCGR1A |  |  |  |  |
| Cytokinins |  | GLUD1 |  |  |  |  |
|  |  | NGF |  |  |  |  |
|  | trans-zeatin riboside | PROZ |  |  |  |  |
|  |  | GPX3 |  |  |  |  |
|  |  | SCN2B |  |  |  |  |
|  |  | GLUD1 |  |  |  |  |
|  | trans-zeatin riboside- 5′-monophosphate | PROZ |  |  |  |  |
|  |  | GPX4 |  |  |  |  |
|  |  | GPX3 |  |  |  |  |
|  |  | NGF |  |  |  |  |
|  |  | GLUD1 |  |  |  |  |
|  |  | PROZ |  |  |  |  |
|  |  | NGF |  |  |  |  |
|  | trans-zeatin riboside-O-glucoside | S100A12 |  |  |  |  |
|  |  | GPX5 |  |  |  |  |
|  |  | GLUD1 |  |  |  |  |
|  |  | NGF |  |  |  |  |
|  |  | PROZ |  |  |  |  |
| Cytokinins | trans-zeatin-9-glucoside | GPX3 |  |  |  |  |
|  |  | GPX5 |  |  |  |  |
|  |  | GLUD1 |  |  |  |  |
|  |  | PROZ |  |  |  |  |
|  |  | SPSB2 | SPRY domain-containing SOCS box protein 2 | Molecular adaptor | Q99619 | 3EMW |
|  | trans-zeatin-O-glucoside | GPX3 |  | | | |
|  |  | SCN2B |  |  |  |  |
|  |  | GLUD1 |  | | | |
|  |  | NGF |  |  |  |  |
|  |  | DDR2 |  |  |  |  |
|  | jasmonic acid | FCGR1A |  |  |  |  |
|  |  | PROZ |  |  |  |  |
|  |  | GLUD1 |  |  |  |  |
| Jasmonates |  | NGF |  |  |  |  |
|  | methyl jasmonate | PROZ |  |  |  |  |
|  |  | CACNA1A |  |  |  |  |
|  |  | FCGR1A |  |  |  |  |
|  |  | GLUD1 |  |  |  |  |
|  |  | NGF |  |  |  |  |
|  | 12-oxo-phytodienoic acid | PROZ |  |  |  |  |
|  |  | GPX3 |  |  |  |  |
|  |  | GPX5 |  |  |  |  |
|  | dinor-12-oxo-phytodienoic acid | GLUD1 |  |  |  |  |
|  |  | NGF |  |  |  |  |
|  |  | PROZ |  |  |  |  |
|  |  | GPX3 |  |  |  |  |
|  |  | RNASE1 | Ribonuclease pancreatic | Hydrolase | P07998 | 2E0L |
|  |  | GLUD1 |  | | | |
|  |  | NGF |  |  |  |  |
|  | gibberellin A1 | DDR2 |  |  |  |  |
|  |  | CLCNKA | Chloride channel protein ClC-Ka | Transport protein | P51800 | 2PFI |
|  |  | FCGR1A |  | | | |
|  |  | GLUD1 |  | | | |
|  |  | NGF |  |  |  |  |
|  |  | FCGR1A |  |  |  |  |
|  | gibberellin A3 | GPX5 |  |  |  |  |
| Gibberellins |  | YES1 |  |  |  |  |
|  |  | GLUD1 |  |  |  |  |
|  |  | SV2A |  |  |  |  |
|  | gibberellin A4 | NGF |  |  |  |  |
|  |  | PROZ |  |  |  |  |
|  |  | DDR2 |  |  |  |  |
|  |  | GLUD1 |  |  |  |  |
|  |  | NGF |  |  |  |  |
|  |  | GPX5 |  |  |  |  |
|  | gibberellin A5 | DDR2 |  |  |  |  |
|  |  | FCGR1A |  |  |  |  |
|  |  | GLUD1 |  |  |  |  |
|  |  | GPX5 |  |  |  |  |
|  |  | NGF |  |  |  |  |
|  | gibberellin A6 | PROZ |  |  |  |  |
|  |  | FCGR1A |  |  |  |  |
| Gibberellins |  | GLUD1 |  |  |  |  |
|  |  | SV2A |  |  |  |  |
|  | gibberellin A7 | NGF |  |  |  |  |
|  |  | DDR2 |  |  |  |  |
|  |  | FCGR1A |  |  |  |  |
|  |  | PROZ |  | | | |
|  |  | GLUD1 |  |  |  |  |
|  | salicylic acid | NGF |  |  |  |  |
| Salicylic acid |  | DDR2 |  |  |  |  |
|  |  | RNGTT |  |  |  |  |
|  |  | GLUD1 |  | | | |
|  |  | PROZ |  |  |  |  |
|  | (+)-strigol | DDR2 |  |  |  |  |
|  |  | NGF |  |  |  |  |
| Strigolactones |  | PPIC | Peptidyl-prolyl cis-trans isomerase C | Isomerase/immunosuppressant | P45877 | 2ESL |
|  |  | GLUD1 |  | | | |
|  |  | PROZ |  |  |  |  |
|  |  | NGF |  |  |  |  |
|  | (+)-strigyl acetate | PGRMC1 |  |  |  |  |
|  |  | FCGR1A |  |  |  |  |
|  |  | GLUD1 |  |  |  |  |
|  |  | PROZ |  |  |  |  |
|  |  | FCGR1A |  |  |  |  |
|  | (+)-orobanchol | NGF |  |  |  |  |
|  |  | DDR2 |  |  |  |  |
|  |  | GLUD1 |  |  |  |  |
|  |  | NGF |  |  |  |  |
|  | (+)-orobanchyl acetate | PROZ |  |  |  |  |
|  |  | DDR2 |  |  |  |  |
| strigolactones |  | CHD1 |  |  |  |  |
|  |  | GLUD1 |  |  |  |  |
|  |  | NGF |  |  |  |  |
|  |  | PROZ |  |  |  |  |
|  | (+)-5-deoxystrigol | YES1 |  |  |  |  |
|  |  | FCGR1A |  |  |  |  |
|  |  | GLUD1 |  |  |  |  |
|  |  | NGF |  |  |  |  |
|  |  | PROZ |  |  |  |  |
|  | sorgolactone | GPX5 |  |  |  |  |
|  |  | DDR2 |  |  |  |  |
