# Supplementary material for: On the human health benefits of microalgal phytohormones: An explorative in silico analysis
Source: Comput Struct Biotechnol J. 2023 Jan 25;21:1092–101. doi: 10.1016/j.csbj.2023.01.032 (PMC9900276; doi:10.1016/j.csbj.2023.01.032)
Supplement: Supplementary file 2 — Supplementary material [file mmc2.docx]

**Supplementary Table S2**

Comparative energy binding scores of the human receptor IMPDH1 to dihydrozeatin or other molecules used as drugs targeting the receptor IMPDH1 (see text)

| **Compound** | **Pubchem CID** | **Score (kcal mol^-1^)** | **RMSD** | **H-bonds** |
| --- | --- | --- | --- | --- |
| dihydrozeatin | 32021 | - 5.5 | 0.0 | 1 |
| mizoribine | 104762 | - 7.3 | 0.0 | 3 |
| mycophenolic acid | 446541 | - 6.3 | 0.0 | 1 |
| ribavirin | 37542 | - 7.0 | 0.0 | 3 |
| tiazofurin | 457954 | - 6.9 | 0.0 | 2 |
